# Canadian Consensus for Treatment of BRAF *V600E* Mutated Pediatric and AYA Gliomas

**DOI:** 10.3390/curroncol31070299

**Published:** 2024-07-16

**Authors:** Craig Erker, Magimairajan Issai Vanan, Valérie Larouche, Liana Nobre, Chantel Cacciotti, Stéphanie Vairy, Shayna Zelcer, Adam Fleming, Eric Bouffet, Nada Jabado, Geneviève Legault, Samuele Renzi, Tara McKeown, Bruce Crooks, Nirav Thacker, Vijay Ramaswamy, Hallie Coltin, Lucie Lafay-Cousin, Sylvia Cheng, Juliette Hukin, Seth Andrew Climans, Mary Jane Lim-Fat, Sarah McKillop, Sarah Lapointe, Mélanie Alves, Julie Bennett, Uri Tabori, Sébastien Perreault

**Affiliations:** 1 Department of Pediatrics, Dalhousie University, Halifax, NS B3H 4R2, Canada; craig.erker@iwk.nshealth.ca (C.E.); bruce.crooks@iwk.nshealth.ca (B.C.); 2 Division of Pediatric Hematology-Oncology, IWK Health Centre, Halifax, NS B3K 6R8, Canada; 3 Pediatric Neuro-Oncology, Division of Pediatric Hematology-Oncology and BMT, Cancer Care Manitoba, 3a Department of Pediatrics and Child Health, Rady Faculty of Health Sciences, University of Manitoba, Winnipeg, MB R3T 2N2, Canada; mivanan@cancercare.mb.ca; 4 Department of Pediatric Hemato-Oncology, CHU de Québec-Université Laval, Quebec City, QC G1V 4G2, Canada; valerie.larouche.med@ssss.gouv.qc.ca (V.L.); samuele.renzi.med@ssss.gouv.qc.ca (S.R.); 5 Department of Pediatrics, University of Alberta, Edmonton, AB T6G 2R3, Canada; liana.nobre@ualberta.ca; 6 Division of Hematology/Oncology, Department of Pediatrics, London Health Sciences Centre & Western University, London, ON N6A 5A5, Canada; chantel.cacciotti@lhsc.on.ca (C.C.); shayna.zelcer@lhsc.on.ca (S.Z.); 7 Department of Pediatrics, Division of Pediatric Hematology, CHU Sherbrooke, QC J1H 5N4, Canada; stephanie.vairy@usherbrooke.ca; 8 McMaster Children’s Hospital, Hamilton, ON L8N 3Z5, Canada; afleming@mcmaster.ca; 9 Department of Pediatrics, University of Toronto, Toronto, ON M5S 1A1, Canada; eric.bouffet@sickkids.ca (E.B.); tara.mckeown@sickkids.ca (T.M.); vijay.ramaswamy@sickkids.ca (V.R.); julie.bennett@sickkids.ca (J.B.); uri.tabori@sickkids.ca (U.T.); 10 Division of Hematology Oncology, The Hospital for Sick Children, Toronto, ON M5G 1E8, Canada; 11 Montreal Children’s Hospital, Montréal, QC H4A 3J1, Canada; nada.jabado@mcgill.ca (N.J.); genevieve.legault4@mcgill.ca (G.L.); 12 Division of Hematology/Oncology, CHEO, Ottawa, ON K1H 8L1, Canada; nthacker@cheo.on.ca; 13 Division of Hematology Oncology, CHU Sainte-Justine, Montréal, QC H3T 1C5, Canada; hallie.coltin.med@ssss.gouv.qc.ca; 14 Alberta Children’s Hospital, Calgary, AB T3B 6A8, Canada; lucie.lafay-cousin@albertahealthservices.ca; 15 B.C. Children’s Hospital, Vancouver, BC V6H 3N1, Canada; sylvia.cheng@cw.bc.ca (S.C.); jhukin@cw.bc.ca (J.H.); 16 Department of Oncology, Western University, London, ON N6A 3K7, Canada; seth.climans@lhsc.on.ca; 17 Odette Cancer Centre, Sunnybrook Health Sciences Centre, University of Toronto, Toronto, ON M5S 1A1, Canada; maryjane.limfat@sunnybrook.ca; 18 Women and Children’s Health Research Institute, University of Alberta, Edmonton, AB T6G 2R3, Canada; sarah.mckillop@albertahealthservices.ca; 19 Division of Neurology, Department of Neurosciences, Montreal, QC H3A 2B4, Canada; sarah.lapointe.med@ssss.gouv.qc.ca; 20 Department of Neurosciences, University of Montreal, Montréal, QC H3T 1J4, Canada; melanie.alves.hsj@ssss.gouv.qc.ca; 21 Division of Child Neurology, CHU Sainte-Justine, Montréal, QC H3T 1C5, Canada

**Keywords:** glioma, pediatric low-grade glioma, high-grade glioma, BRAF V600E mutation, BRAF inhibitor, MEK inhibitor

## Abstract

**Background:** The treatment of BRAF V600E gliomas with BRAF inhibitors (BRAFis) and MEK inhibitors (MEKis) has been increasingly integrated into clinical practice for pediatric low-grade gliomas (PLGGs) and pediatric high-grade gliomas (HGGs). However, some questions remain unanswered, such as the best time to start targeted therapy, duration of treatment, and discontinuation of therapy. Given that no clinical trial has been able to address these critical questions, we developed a Canadian Consensus statement for the treatment of BRAF V600E mutated pediatric as well as adolescent and young adult (AYA) gliomas. **Methods**: Canadian neuro-oncologists were invited to participate in the development of this consensus. The consensus was discussed during monthly web-based national meetings, and the algorithms were revised until a consensus was achieved. **Results**: A total of 26 participants were involved in the development of the algorithms. Two treatment algorithms are proposed, one for the initiation of treatment and one for the discontinuation of treatment. We suggest that most patients with BRAF V600E gliomas should be treated with BRAFis ± MEKis upfront. Discontinuation of treatment can be considered in certain circumstances, and we suggest a slow wean. **Conclusions:** Based on expert consensus in Canada, we developed algorithms for treatment initiation of children and AYA with BRAF V600E gliomas as well as a discontinuation algorithm.

## 1. Introduction

Gliomas are the most frequent CNS tumor in pediatric, adolescent, and young adult (AYA) patients [1]. While a complete resection can be achieved for some patients, the majority will need systemic therapy to prevent further progression [2]. Standard systemic treatment for pediatric low-grade gliomas (PLGGs) has traditionally been chemotherapy with either carboplatin/vincristine or weekly vinblastine [3,4]. Unfortunately, many patients will progress despite this treatment approach (50%) and a second line of therapy will be needed [3,4]. 

High-grade gliomas (HGGs) represent just under 10% of the central nervous system (CNS) tumors in the pediatric and AYA population [5,6]. Most pediatric HGGs are diffuse midline gliomas. Standard treatments for pediatric HGGs include focal radiation therapy with or without adjuvant or concurrent chemotherapy. Progression-free survival (PFS) and overall survival (OS) are generally poor for HGG.

Over the last decade, it has been well established that over 90% of PLGGs have an alteration of the MAPK pathway [7]. The most frequent alteration is the *KIAA1549-BRAF* fusion (35%) followed by the BRAFV600E (17%) mutation and those related to germline neurofibromatosis-1 (*NF1*) (17%) [7,8]. It has been reported that PLGGs with the BRAF V600E mutation have a worse prognosis compared with other alterations such as *NF1* and *KIAA1549-BRAF* fusion [9]. The BRAF V600E mutation can be targeted with specific inhibitors, and the efficacy of BRAF inhibitors (BRAFis) with or without the combination with MEK inhibitors (MEKis) has been reported in several case reports, case series, and clinical trials [10,11,12,13,14,15]. Recently, a randomized clinical trial demonstrated that BRAFis combined with MEKis improved the overall response rate and duration of response with fewer adverse events when compared with the carboplatin/vincristine regimen [10]. 

A significant percentage (10%) of pediatric and AYA HGGs have the BRAF V600E mutation [16]. The PFS and OS appear to be better than other subtypes of HGG, but the best treatment approach has not been determined. Hargrave et al. reported an overall response rate of 56% and a median overall survival of 32.8 months when treated with a combination of dabrafenib and trametinib in the recurrent setting [11]. Other case reports, case series, and studies have supported these observations (review [14]).

The treatment of BRAF V600E mutated gliomas with BRAFis and MEKis is being increasingly integrated into the clinical practice for PLGG and pediatric HGG. However, some questions remain unanswered, such as when to initiate treatment, duration of treatment, and approach to treatment discontinuation. Given that no clinical trial is currently prepared to address these specific and critical questions, we developed a Canadian Consensus for the Treatment of BRAF V600E Mutated Pediatric Gliomas.

## 2. Methods

Canadian pediatric and adult neuro-oncologists and pediatric nurse practitioners specialized in neuro-oncology were invited to participate in the development of this consensus. A survey regarding the practice and management of gliomas with BRAFV600E mutation was sent. Results from this survey initiated the discussion, and a first consensus guidelines draft was developed. Discussions continued during monthly web-based national CNS tumor rounds. Once the first consensus treatment algorithm was established, it was shared with participants by email. The algorithm and suggestions were discussed by email and during national rounds until a consensus was achieved.

## 3. Results

A total of 26 participants (21 pediatric neuro-oncologists, four adult neuro-oncologist, and one nurse practitioner) were involved in the development of the algorithms. This represents more than 75% of pediatric neuro-oncologists practicing currently in Canada. The first discussions took place in October 2021 and the consensus algorithms were completed in September 2022. The algorithms were reviewed and approved in July 2023. 

**Figure 1:**
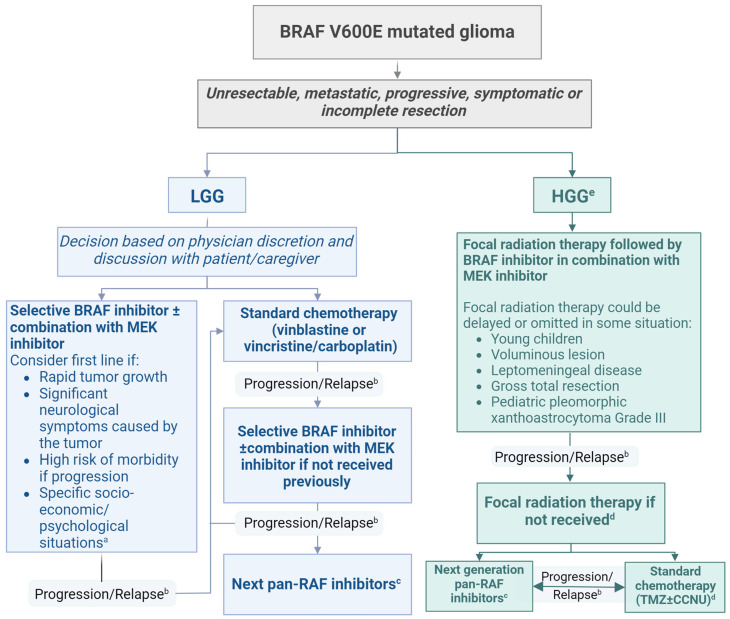
Treatment algorithm for pLGG and HGG with BRAF V600E mutation. a: including inability to receive weekly IV treatment due to long distance between home and hospital or high psychological impact of frequent visits for the child or family; b: consider resection or biopsy if progression despite optimal treatment; c: on clinical trial/study if possible; d: consider continuing BRAF inhibitors and MEK inhibitors concurrently with radiation therapy or standard chemotherapy in cases where rapid progression could be associated with neurological deterioration. Caution should be taken since these combinations have not been studied in the setting of a clinical trial for CNS tumors; e: please refer to the discussion section for specific discussion on HGG.

**Figure 2:**
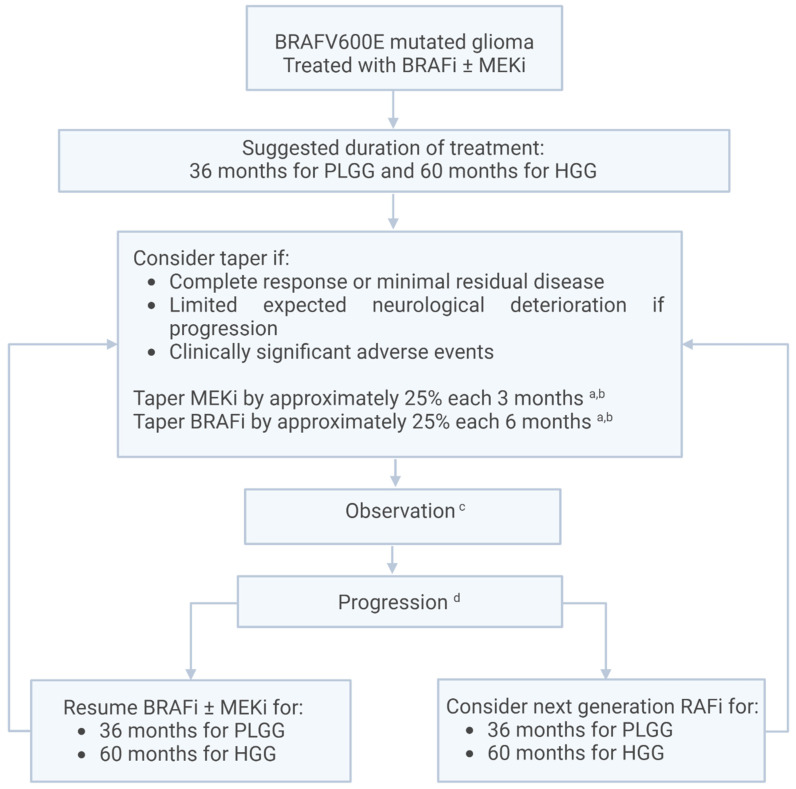
Discontinuation algorithm for PLGG and HGG with BRAFV600E mutation. Suggested surveillance MRI frequency during treatment: every 3 months for 2 years, and every 6 months after if stable and depending on clinical evolution. a: if the dose cannot be tapered by approximately 25% every three months due to tablet size/formulation. Consider alternate dosing (3 days on/one day off, 1 day on/1 day off, 1 day on/3 days off). b: MEKi could be tapered before BRAFi. c: taper 1 month prior to the next planned surveillance MRI. d: consider resection or biopsy if progression occurs.

Two treatment algorithms were developed, one for the initiation of treatment (Figure 1) and one for the discontinuation of treatment (Figure 2). 

Example of suggested taper:

Timepoint 0 months—BRAFi 100%; MEKi 100%
Timepoint 3 months—BRAFi 100%; MEKi 75%
Timepoint 6 months—BRAFi 75%; MEKi 50%
Timepoint 9 months—BRAFi 75%; MEKi 25%
Timepoint 12 months—BRAFi 50%; MEKi 0
Timepoint 15 months—BRAFi 50%; MEKi 0
Timepoint 18 months—BRAFi 25%; MEKi 0
Timepoint 21 months—BRAFi 25%; MEKi 0
Timepoint 24 months—BRAFi 0; MEKi 0

We suggest continuing with surveillance MRI at least every 3 months once off drugs for 1 year then as per institutional standard.

## 4. Discussion

Our group developed a consensus for the treatment of PLGG and HGG with the BRAF V600E mutation. We also include a proposed discontinuation of the treatment algorithm. These guidelines are based on current expert opinions and could be used as a reference to guide therapeutic decisions. However, we acknowledge that each patient and situation is different and that other considerations, such as age, tumor location, and previous treatments, can modify clinician management of individual patients. 

Rapid tumor growth, significant neurological symptoms caused by the tumor, and high risk of morbidity if progression occurs should prompt the initiation of targeted therapy. In some cases, where the evolution of the tumor is unknown, it is possible to observe with close follow-up surveillance MRI. Several publications and a recent randomized study suggest that BRAFis and MEKis are more efficacious and better tolerated than chemotherapy for PLGG with the BRAF V600E mutation and that targeted therapy can be used as first-line systemic treatment [10,11,12,13,14,15]. Our group felt that, given the fact that some questions remain regarding the duration of treatment with targeted therapy and outcome at discontinuation, chemotherapy should not be removed from the treatment options and that a thorough discussion with the family is crucial. Specific socio-economic situations, such as patients living far away from hospitals, can impact the decision since it is generally easier to administer oral drugs at home with BRAFis with or without MEKis than weekly intravenous (IV) chemotherapy infusions. Dealing with frequent IV treatment and weekly hospital visits can also be challenging psychologically for some patients. 

Even though the most recent studies used the combination of BRAFis and MEKis, and some studies suggest that the combination provides better disease control and decreases adverse events [17,18], our group felt it was reasonable to use BRAFis as monotherapy for some patients with BRAF V600E mutant gliomas. In some instances, monotherapy could facilitate compliance and reduce costs. In addition, MEKis can be added to the treatment if there is tumor progression on monotherapy with BRAFis.

Most studies have used dabrafenib (BRAFi) and trametinib (MEKi) for the treatment of gliomas [10,19]. However, similar responses have been reported with other BRAFis, such as vemurafenib [20,21,22]. Currently, no data support that a specific BRAFi or MEKi is more effective or better tolerated than another. Drug access (in most parts of the world) is probably one of the most important factors that would currently guide the use of one targeted drug over another. If adverse events lead to discontinuation of treatment, another BRAFi/MEKi could be considered. Our group currently suggests switching to a pan-RAFi in cases of progression given that the difference in efficacy is probably minimal between two different BRAFis or MEKis.

If a patient’s tumor progresses after standard chemotherapy, BRAFi ± MEKi is recommended. If feasible, and if molecular testing is accessible, we suggest a biopsy after progression to better understand the evolution of the lesion including possible new resistant mutations with reactivation of the RAS/RAF/ERK pathway (review [23]). If the patient received BRAFi ± MEKi and subsequently progressed, clinical trials should be considered and new drugs, such as tovorafenib or plixorafenib (for patients ≥ 12 years old), might be considered [24,25,26]. 

Maximal safe resection followed by focal radiation therapy with concurrent, and with or without adjuvant, temozolomide is currently the standard treatment for HGG [27]. Even though treatment outside of these guidelines and access to BRAFi ± MEKi might be challenging (especially in adults), we suggest that patients with HGG and the BRAFV600E mutation should receive focal radiation therapy followed by targeted therapy. We acknowledge that there is currently no randomized trial comparing the efficacy of chemotherapy to targeted therapy but, given the generally poor outcomes of patients treated with temozolomide and the high response rate and favorable safety profile of BRAFi ± MEKi [19], we believe that targeted therapy should be considered upfront. 

In some instances, radiation could be delayed or omitted. It may be reasonable to save radiotherapy for recurrence after BRAFi ± MEKi in cases where radiation toxicity would be highest, such as in young children, in patients with very large tumors, or in patients with leptomeningeal disease who would require large radiation fields. It may also be reasonable to save radiotherapy for recurrence in cases where the HGG can be expected to behave less aggressively, such as in patients with gross total resection. There was controversy, but some argued that radiation could be withheld for pediatric patients with grade 3 pleomorphic xanthoastrocytoma (PXA) since these patients probably have a better prognosis than other non-PXA HGG [28]. Given that the median time to respond for HGG is relatively short and the clinical status of the patient could be improved, we consider it reasonable to use concurrent targeted therapy treatment during radiation therapy. Initial reports suggest that BRAFis can increase the toxicity of radiation therapy with more cutaneous adverse effects [29], but recent studies for both non-CNS and CNS tumors have suggested that it is well tolerated [30,31]. All these cases should be presented at tumor rounds followed by a comprehensive discussion with the patient and family. 

Currently, there are limited data on the duration of treatment and outcomes once targeted therapy is discontinued for patients with gliomas and the BRAF V600E mutation. Case reports and case series have reported dramatic rapid progression for some patients upon abrupt discontinuation of targeted therapy [32,33]. However, long-term treatment is likely to be associated with adverse events, decreased QOL, and financial impact, although these factors are poorly studied at present. Very long-term late effects are currently unknown. Discontinuation of treatment could be considered for specific patients with complete response or minimal residual disease and limited expected neurological deterioration in the event there is rapid recurrence or progression. Surgery should be considered in patients who have the possibility of achieving a gross total resection following partial response to treatment, as this would potentially be curative. Based on clinical experience and consensus, we currently suggest treating with BRAFi ± MEKi for a total of 36 months for PLGG and 60 months for HGG. Discontinuation should be considered sooner if there are clinically significant adverse events.

Since discontinuation of treatment can be associated with rapid clinical and radiological progression, we suggest tapering one drug at a time, starting with MEKis by approximately 25% every 3 months, and the BRAFis by 25% every 6 months. The consensus was to taper MEKis first given the fact that gliomas with the BRAF V600E mutation can be treated effectively with BRAFi monotherapy. A completed tapering and stopping of the targeted therapy would take 24 months based on our suggested schedule, but we acknowledge that the speed of the taper must be adapted to individual cases. We recommend radiological surveillance every 3 months with MRI. Ideally, each taper step would be performed 1 month prior to the scheduled MRI to identify any possibly rapid progression of the tumor while limiting the number of imaging exams. 

If a significant radiological or clinical progression is identified, we suggest reverting back to the last effective dose prior to progression. If further progression occurs, the patient should receive the original full dose or consider switching to a pan-RAFi. Surveillance without increasing the doses could also be considered in the context of a progression that remains smaller than the baseline, is not associated with clinical symptoms, and will have a limited impact if further progression occurs. Transitory rebound phenomena can be seen based on our experience, and dose escalation is not always indicated. 

## 5. Conclusions

Based on expert consensus in Canada, we developed both an algorithm for the initiation of treatment for children and AYA with BRAF V600E gliomas as well as a discontinuation of the treatment algorithm. These guidelines could help in the decision-making and management of patients harboring this mutation, which is particularly sensitive to targeted therapy. We are hopeful that this work will raise new questions and help with the development of new clinical trials.
